# Microbial Source-Tracking Reveals Origins of Fecal Contamination in a Recovering Watershed

**DOI:** 10.3390/w11102162

**Published:** 2019-10-17

**Authors:** Hyatt Green, Daniel Weller, Stephanie Johnson, Edward Michalenko

**Affiliations:** 1 Department of Environmental and Forest Biology, College of Environmental Science and Forestry, State University of New York, New York, NY 13210, USA; 2 Department of Biostatistics and Computational Biology, University of Rochester, 265 Crittenden Blvd., Rochester, NY 14642, USA; 3 Onondaga Environmental Institute, 5795 Widewaters Pkwy, Syracuse, NY 13214, USA

**Keywords:** enterococcus, stormwater management, *Bacteroides*, HF183, fecal coliforms, agricultural water quality, Onondaga Lake

## Abstract

Fecal contamination of waterbodies due to poorly managed human and animal waste is a pervasive problem that can be particularly costly to address, especially if mitigation strategies are ineffective at sufficiently reducing the level of contamination. Identifying the most worrisome sources of contamination is particularly difficult in periurban streams with multiple land uses and requires the distinction of municipal, agricultural, domestic pet, and natural (i.e., wildlife) wastes. Microbial source-tracking (MST) methods that target host-specific members of the bacterial order *Bacteroidales* and others have been used worldwide to identify the origins of fecal contamination. We conducted a dry-weather study of Onondaga Creek, NY, where reducing fecal contamination has been approached mainly by mitigating combined sewer overflow events (CSOs). Over three sampling dates, we measured in-stream concentrations of fecal indicator bacteria; MST markers targeting human, ruminant, and canine sources; and various physical–chemical parameters to identify contaminants not attributable to CSOs or stormwater runoff. We observed that despite significant ruminant inputs upstream, these contaminants eventually decayed and/or were diluted out and that high levels of urban bacterial contamination are most likely due to failing infrastructure and/or illicit discharges independent of rain events. Similar dynamics may control other streams that transition from agricultural to urban areas with failing infrastructure.

## 1. Introduction

Improper disposal or inadequate containment of fecal waste has well-documented, detrimental effects on human [1] and environmental health [2]. Runoff from agricultural and residential areas, as well as aging infrastructure (e.g., septic systems), sewer overflows, and illicit discharges have all been identified as potential sources of fecal contamination. Differentiating the degree (i.e., frequency and volume) of contribution from each source is the first step in determining how to best mitigate fecal contamination for a given waterbody. In areas with combined sewer systems, urban creeks receive human fecal contaminants from combined sewer overflows (CSO) during rain events only, while failing infrastructure and illicit discharges contribute contaminants during both wet and dry weather. Further complicating matters, urban stream reaches may also receive contaminants from rural sources upstream, as well as from domestic pets and wildlife in both the rural and urban reaches. This multiplicity of potential fecal contamination sources can make identifying, prioritizing, and remediating major inputs of bacterial contamination particularly challenging.

Traditional methods of identifying sources of fecal contamination often include spatial analysis of fecal indicator bacteria (FIB; e.g., *E. coli* or *Enterococcus*) levels. Although this approach has been successfully used to identify major sources of contaminants in urban streams [3], it is limited by the generalist nature of FIBs, which are found in high abundance in human, ruminant, wildlife, and other sources of fecal contaminants [4,5]. Therefore, enumeration of FIB in ambient waterbodies, while useful for gauging compliance to water quality standards and overall levels of fecal contamination, is not an ideal approach to identify specific sources of fecal contaminants in areas where noncompliance is the norm. Microbial source-tracking (MST) methods have been developed to target members of *Bacteroidales* or other bacteria that are specific to human [6–10], ruminant [11,12], or other sources [13–16], which allow quantification of source-specific contaminants from ambient water samples.

In this study, we applied molecular (both MST and *Enterococcus*) and traditional techniques to routinely noncompliant Onondaga Creek (Onondaga County, NY, USA) during three dry-weather sampling dates to identify patterns of contamination across the rural–urban gradient. Despite significant investment in mitigating contaminant inputs from CSO events, the creek routinely exceeds regulatory limits of FIB even during dry weather, suggesting the need for management strategies that are targeted to specific fecal sources. The first step in developing these strategies is understanding what fecal contamination sources are present in Onondaga Creek, and how these vary within the watershed. Broadly, we hypothesized that ruminant MST markers would be found at significant levels in downstream, urban areas because of high bacterial loading to stream tributaries due to a high proportion of agricultural land use and relatively rapid stream transport over a distance of about 40 km from rural to urban areas. Under this general hypothesis, the specific aims of our study were to determine (i) if levels of FIB and MST markers were significantly different at urban and rural sites; (ii) if FIB levels were significantly associated with MST markers levels or detection; and (iii) if this relationship differed between urban and rural sites. Generally, results show that ruminant contaminants were introduced upstream but were sparse downstream, indicating that contaminant inputs upstream are likely not significant contributors to total FIB loads in downstream urban areas during dry periods. Rather, high levels of human contaminants were identified during dry weather, suggesting that failing infrastructure and/or illicit discharges are a persistent, major contributor of fecal contamination to Onondaga Creek independent of wet-weather discharges likely causing routine violations of water quality standards during the dry season.

## 2. Materials and Methods

### 2.1. Study Site

**Figure 1.**
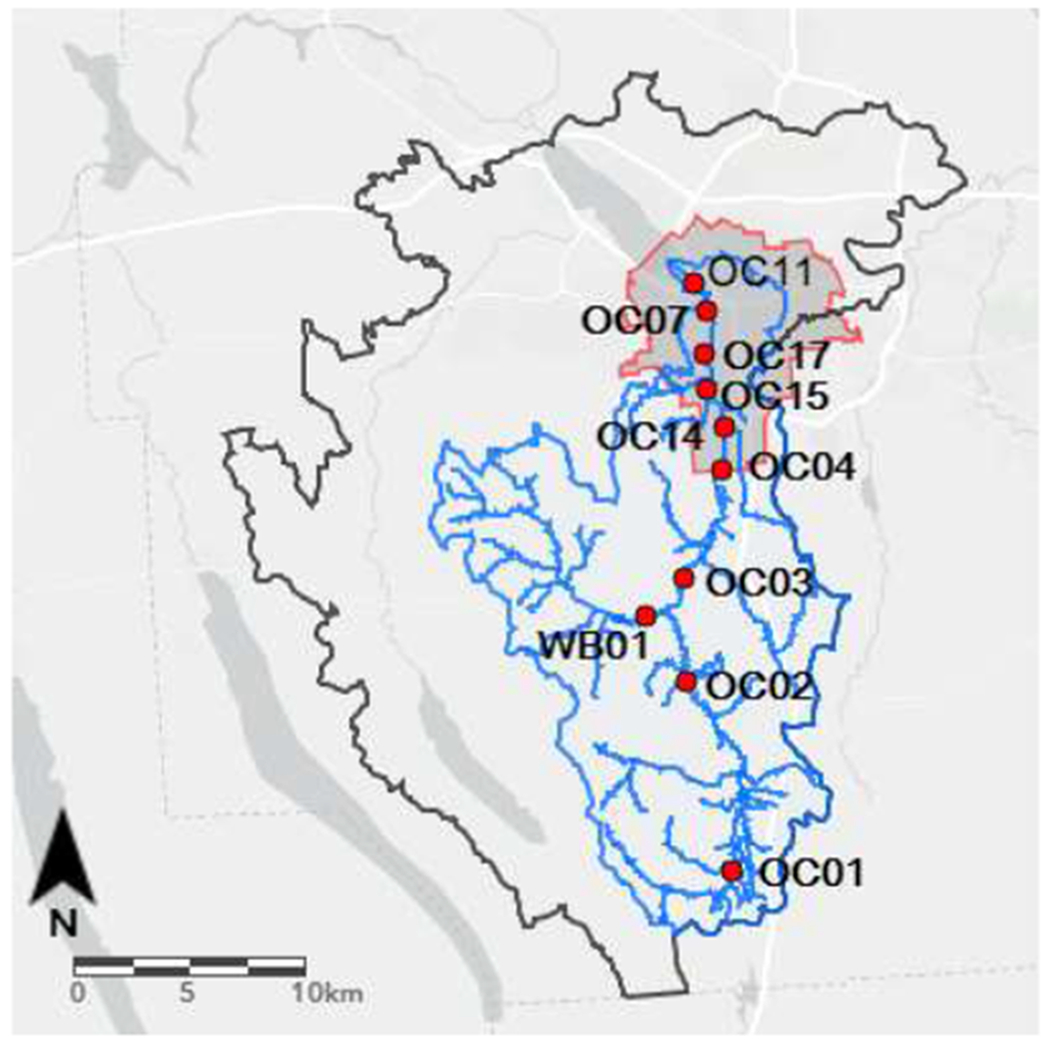
Map of sampling sites within Onondaga Lake (grey border) and Onondaga Creek (blue border) watersheds in relation to the City of Syracuse (red border).

Onondaga Creek has the second largest drainage area (298 km^2^) in the Onondaga Lake watershed and contributes the greatest surface water inflow. The creek is approximately 26.7 mi (43 km) in length and descends more than 0.19 mi (0.30 km) from its headwaters [17]. Land use is predominantly forest (50%) and agriculture (31%), with approximately 19% considered developed [18]. The stream follows a rural-urban gradient from the southern headwaters in Tully, NY to the northern outlet to Onondaga Lake in the City of Syracuse (Figure 1). Onondaga Lake receives approximately 35% of its surface water inflow and upwards of 12% of annual fecal coliform loads from Onondaga Creek [19].

Sampling sites were selected based on ease of access (e.g., bridges), many of which were sampling sites used in previous monitoring efforts. Percent land use was determined for each basin using the Spatial Analyst ToolPak in ArcGIS10 (ESRI, Redlands, CA, USA). These estimates were used to categorize locations as either “rural”, which included agricultural land use; or “urban,” which included low and high intensities of development.

### 2.2. Sample Collection, Storage, and Processing

Sampling was conducted under dry weather conditions on 6 July, 20 July, and 3 August 2015. Dry weather conditions were defined as a minimum of two days with little or no precipitation in Syracuse. Specifically, sampling was initiated only when no more than 2 mm of rain had fallen during the preceding 48-h period.

One-liter grab samples were collected from the centerline of the stream using a Coli Sampler [3] just below the water surface. All sampling (30 samples total) was performed in a downstream to upstream manner to prevent sampling the same slug of water. Two subsamples were collected from a single grab sample, one stored in a 125 mL bottle; the other in a 500 mL Nalgene™ plastic bottle. Samples were stored on-ice for delivery to the respective laboratories within no more than five hours after collection.

Five-hundred milliliter samples for MST were taken to SUNY-ESF and filtered on 0.4-μm pore-size 47-mm polycarbonate filters (Whatman, Chicago, IL, USA). All samples were filtered within 8 h of collection. Rolled filters were stored directly in bead tubes supplied in the DNA-EZ RW02 extraction kits (GeneRite, North Brunswick, NJ, USA) which were then stored at −80 °C until DNA extraction. DNA extraction was performed with the DNA-EZ RW02 extraction kit following the manufacturer’s procedure. DNA was eluted in 100 μL and stored at −20 °C until quantitative polymerase chain reaction (qPCR) analysis. Fecal coliforms were enumerated from the 125 mL sample by membrane filtration using standard method 9222 D-97 [20].

### 2.3. Physical-Chemical Parameters

Water quality parameters were measured and recorded in the field at each sampling location for all sampling events using a YSI 650 MDS handheld device (YSI Inc., Yellow Springs, OH, USA) equipped with either a 6600 or 6820-V2 (YSI) multi-parameter water quality monitoring probe. Measured water quality parameters included pH, dissolved oxygen, specific conductivity, temperature, salinity, and turbidity.

### 2.4. Microbial Source-Tracking qPCR Assays

Previously validated probe-based MST methods (Table 1) were used to determine the major sources of fecal contamination in each water sample. In addition to MST markers, enterococci markers were also enumerated to provide an additional measure of overall fecal contamination in each sample. Briefly, 25 μL qPCR reactions consisted of 1X Environmental Master Mix (Life Technologies, Carlsbad, CA, USA), assay-specific oligonucleotide concentrations (Table 1), and two microliters DNA extract. Triplicate reactions for each sample were run on a QuantStudio 3 (Life Technologies) using default cycling conditions as follows: 50 °C for 2 min, 95 °C for 10 min, and 40 cycles of 95 °C for 15 s and 60 °C for 1 min. Before data export, the baseline was set to ‘automatic’ and the fluorescence threshold to 0.03.

### 2.5. qPCR Quality Assurance and Quality Control

Blanks using distilled water were created during filtration and DNA extraction. At least three no template controls (NTCs) were included with each qPCR run. Standard curves used to convert Ct (fractional cycle values at the predefined threshold) values to copy number per well were generated using synthetic double-stranded DNA standard (gBlock®, IDT, Coralville, IA, USA) containing the target sites of all assays used. Samples in which any of the three Ct values were greater than the standard curve intercept for each assay were considered below the limit of detection (LOD) and excluded from analysis. Effects of qPCR inhibition were screened for using kinetic outlier detection using standard curves as reference (i.e., “uninhibited”) reactions [23,24].

### 2.6. Statistical Methods

All statistical analyses were performed in R version 3.6.0 [25]. To address the fact that some samples were below the LOD for the various microbial targets, two approaches were used. For targets where approximately half of samples were below LOD (HF183 and Rum2Bac), two variables were generated for use as explanatory factors in downstream analyses: (i) Detection or failure to detect the given target in a sample; and (ii) log10 concentration of the target was used. For the latter variable, non-detects were assigned the value of log10(0.5 × LOD) or 1.69897 (LOD = 50 markers/100 mL). For targets where only one sample was below the LOD (fecal coliforms and *Enterococcus* markers), the log10 concentration of the target was used. Non-detects were assigned the value of log10(0.5 × LOD) or 1.69897 for *Enterococcus* (LOD = 50 markers/100 mL) and 0.69897 for fecal coliforms (LOD = 10 CFUs/100 mL).

The leaflet package [26] was used to visualize the average concentration of FIBs and MST markers at each site over the three sampling visits, as well as frequency of MST marker detection at each site, and average values for physical-chemical water quality parameters at each site (Maps S1 and S2).

To examine how microbial and physical–chemical water quality varied over the three sampling visits and within the watershed, generalized linear mixed models (GLMM) were developed using the lme4 package [27]. Specifically, to examine how water quality varied over time, GLMMs were developed where the water quality parameter was the outcome, visit was included as a categorical fixed effect (reference-level was the first visit on 6 July 2015), and site ID was included as a random effect. To examine how water quality varied spatially, separate models were developed with either latitude (as a proxy for amount of developed land around a site) or land use category (i.e., urban/ rural; rural was the reference level) as the fixed effect. In the spatial model, visit was included as a fixed effect and site ID was included as a random effect. Microbial water quality variables included log10 *Enterococcus* marker concentration, log10 fecal coliform concentration, log10 MST marker concentrations, and if each MST marker was detected (i.e., as a binary categorical variable). Physical–chemical water quality variables included dissolved oxygen levels (mg/L), log10 conductivity (μS/cm), log10 turbidity (NTU), pH, salinity (ppt), and water temperature (°C). Due to the number of samples that were below the LOD for human and ruminant markers, hurdle models were developed when these were the outcome. The hurdle model consisted of two separate models: (i) Generalized linear mixed model, where the outcome was detection of the marker in a given sample (binary; logit link function used); and (ii) a general linear mixed model where the outcome was the log10 concentration of the marker in samples where the marker was above LOD.

Similarly, to identify associations between microbial and physical–chemical water quality parameters, separate GLMMs were also developed. GLMMs were used in addition to conventional correlation measures (e.g., Spearman’s correlation, Figure S1) to account for pseudoreplication through explicit inclusion of spatial (i.e., site ID as a random effect) and temporal (i.e., visit as a fixed effect) parameters in the models (Table S1).

Finally, GLMMs were also developed to characterize the relationship between FIBs and MST markers, and to determine if the relationship between FIBs and MST markers was different in rural versus urban areas. For each combination of outcome (i.e., log10 fecal coliform concentration and log10 *Enterococcus* concentration) and MST marker (i.e., HF183 detection, log10 HF183 concentration, Rum2Bac detection, and log10 Rum2Bac concentration), three models were developed. The first model was developed to determine if there was an association between the outcome and MST marker; the model included the given MST marker variable and visit as fixed effects and site ID as a random effect. The second model (or “base model”) included the given MST marker variable, land use (i.e., was the site urban or rural, rural was the reference-level), and visit as fixed effects and site ID as a random effect. The third model (or “expanded model”) included the same covariates as the base model as well as an interaction between the given MST marker variable and land use. A likelihood ratio test was used to determine if the expanded model fit the data better than the base model; a significant result for the likelihood ratio test indicated the relationship between the given MST marker and the outcome was different for urban compared to rural sites.

## 3. Results

### 3.1. Physical–Chemical Parameters

Physicochemical measurements from this study are, for the most part, in agreement with extensive surveys conducted on the creek in recent years ([3,28,29]; Table 1). pH significantly decreased in the downstream direction as a function of latitude (*p* < 0.001; Table 2; Map S1) presumably due to surficial changes in geology; with the upper, rural watershed comprised of alkaline limestone bedrock, and the lower, urban reaches predominantly lined with concrete and relatively disconnected from the natural geology of the lower watershed [30]. Dissolved oxygen also decreased significantly in the downstream direction (*p* = 0.008; Table 2; Map S1), which may be due to increased biological and chemical oxygen demand. While log10 conductivity (*p* = 0.082) and salinity (*p* = 0.086) increased numerically in the downstream direction, these trends were not significant. Natural salt springs in the upper and lower reaches of the watershed, as well the use of road salt during winter months, have been shown to negatively impact stream salinity well into the summer months [28,29]. With the exception of log10 conductivity all physical–chemical parameters that were measured varied significantly over the three sampling visits, suggesting that unmeasured temporal factors (e.g., weather) were influencing water quality over the course of the study (Table 2). The relationship between physical–chemical water quality parameters are reported in Table S1.

### 3.2. Fecal Indicator Bacteria Concentrations

**Figure 2.**
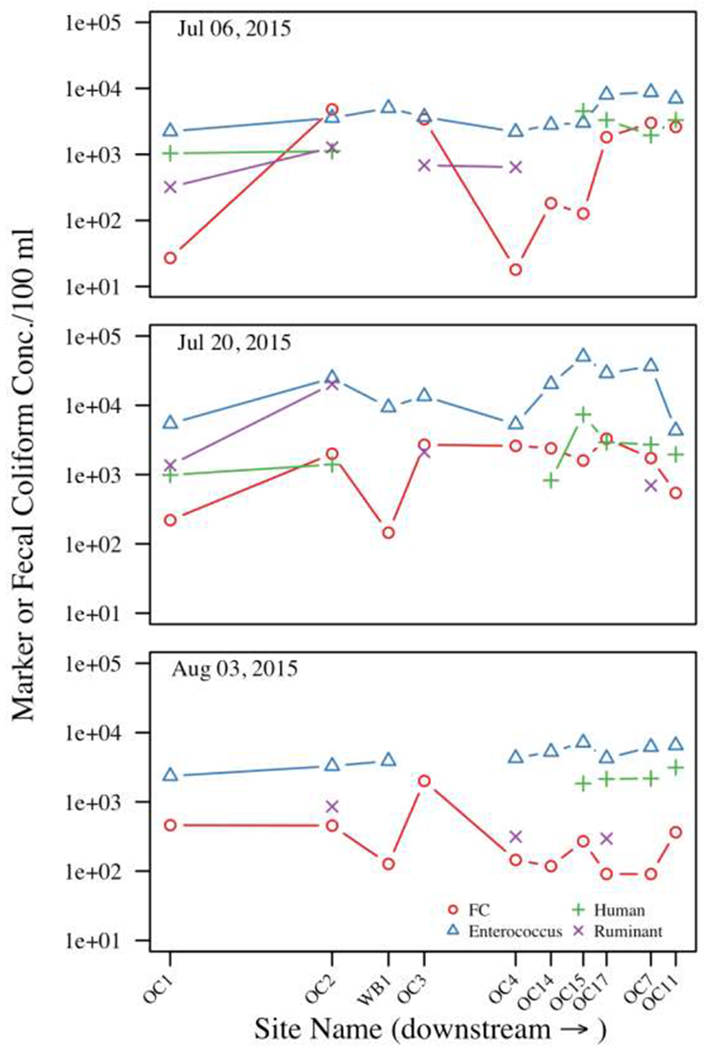
Fecal indicator and molecular marker concentrations during the study period. Symbols indicate MST marker concentrations while segments connect adjacent sites with detectable values of the same marker.

Fecal coliforms levels exceeded the LOD of 10 CFUs/100 mL in 97% (29/30) of samples, and were detected at all ten sites. Due to the small number of samples collected from each site (N = 3), we were unable to determine if fecal coliform concentration at each site met the NY State standard (geometric mean < 200 CFU/100 mL based on 5 samples collected over a month). However, at seven of the ten sites, fecal coliform levels exceeded a geometric mean of 200 CFU/100 mL (Table 2). Fecal coliforms averaged 1.3 × 10^3^ (±1.3 × 10^3^) CFU/100 mL across the entire study with no significant difference between rural and urban sites (*p* = 0.823) according to GLMM (Table 3). Sites OC2 and OC3 had the two highest geometric mean fecal coliform values of all sites. While there were substantial increases in fecal coliform concentration at sites OC1 to OC2 between the first two sampling visits, we did not see a similar trend at other sites. According to GLMM, the log10 concentration of fecal coliforms was, on average, −0.6 (95% Confidence Interval (CI) = −1.12, −0.01; *p* = 0.048) and −0.7 log10 (95% CI = −1.24, −0.13; *p* = 0.016) lower at visits 2 and 3 compared to visit 1. Fecal coliform levels in samples collected from the west branch of the creek (site WB1) were routinely lower than in samples collected from the main branch, which may indicate that the West Branch may dilute fecal coliform concentrations in the main branch. Fecal coliforms consistently decreased from OC3 to OC4, indicating a lack of inputs in this segment and/or significant decay (Figure 2). However, despite these qualitative differences, we did not find evidence of a significant difference in log10 fecal coliform levels at urban compared to rural sites (*p* = 0.823), or as one moved S to N (*p* = 0.394) or E to W (*p* = 0.975) within the watershed using GLMM.

*Enterococcus* marker concentrations loosely followed similar trends as fecal coliforms, albeit at higher concentrations (Figure 2). *Enterococcus* averaged 1.0 × 10^4^ (±1.2 × 10^4^) markers/100 mL across the entire study and was detected in all samples except one (OC3 on Aug 03, 2015). In contrast to fecal coliforms, *Enterococcus* concentrations were approximately 0.36 log10 higher at urban sites compared to rural sites (95% CI = 0.02, 0.70; *p* = 0.041; Table 3) according to GLMM. Like fecal coliform levels, *Enterococcus* marker concentrations were approximately 0.6 log10 (95% CI = −0.96, −0.14; *p* = 0.009) and 0.7 log10 (95% CI = −1.14, −0.32; *p* < 0.001) lower at visits 2 and 3 compared to visit 1. The relationship between FIBs and physical-chemical water quality parameters are reported in Table S1.

### 3.3. Molecular Marker Concentrations

Canine, human, and ruminant fecal markers were detected in 3% (1/30), 57% (17/30), and 37% (11/30) of samples, respectively. In samples where the markers were detectable (LOD 50 markers/100 mL) the concentration of human and ruminant fecal markers ranged between 825 and 7364 (Geometric Mean = 2133; Median = 2141) and between 294 and 20,326 (Geometric Mean = 951; Median = 696), respectively (Table 2). Ruminant markers were detected in 58% (7/12) of samples collected from rural sites and 22% (4/18) of samples collected from urban sites, while human markers were detected in 33% (4/12) of samples collected at rural sites and 72% (13/18) of samples collected at urban sites. Half of all ruminant-positive samples collected from urban sites were collected from OC4 (2/4), which is at the edge of the rural–urban gradient. On both sampling visits in July, human markers were found at the southernmost sites, OC1 and OC2. Human markers were detected at all sampling visits in samples from the four most-downstream sites (OC15, OC17, OC7, and OC11), which are in the City of Syracuse and had the highest proportion of developed land uses (Map S2). Ruminant markers were detected from one site, OC2, on all sampling visits, which was also the source of the sole dog marker positive sample collected at a rural site (OC2; July 20,2015; 5.6 × 10^3^ markers/100 mL). Human markers were never detected at OC3, OC4, or WB1; while ruminant markers were never detected at WB1, OC11, OC14, or OC15.

Qualitatively, there also appears to be a spatial pattern in human and ruminant marker detection, with human markers being repeatedly detected in urban samples and intermittently detected in rural samples; and ruminant markers being repeatedly detected in rural samples (and OC4 which is at the urban-rural interface) and intermittently detected in urban samples (Map S2; Figure 2). However, these associations only appear to be significant for the human markers. The log10 concentration of human markers in human-marker-positive samples was 0.37 log10 higher at urban sites compared to rural sites (95% CI = 0.04, 0.69; *p* = 0.030). The likelihood of detecting human markers was also significantly higher for urban sites compared to rural sites (*p* =< 0.001). Neither ruminant marker detection (*p* = 0.101) nor the log10 concentration of ruminant markers in ruminant-marker-positive samples (*p* = 0.953) were significantly associated with land use. However, this could be due to absence of a true association or the small number of samples (e.g., there were only eleven ruminant-marker-positive samples). In fact, when samples where ruminant marker concentrations below the LOD were assigned a marker concentration of log10(0.5 × LOD), there appeared to be a significant association between log10 ruminant marker concentration and land use (Effect Estimate = −0.75; 95% CI = −1.49, −0.02; *p* = 0.046). For both markers, it appears that the majority of human-marker-positive samples and ruminant-marker-negative samples were above 43 degrees latitude while most human-marker-negative samples and ruminant-marker-positive samples were below 43 degrees (Table 2).

There appears to be a temporal trend in MST marker detection, with human and ruminant markers being detected at more sites on the first and second sampling visits compared to the third (Figure 1). However, there does not appear to be a significant association between sampling visit and the log10 concentration of human markers in human marker positive samples (*p* = 0.496), nor the detection of human (*p* = 0.696) and ruminant (*p* = 0.825) (Table 3). However, we did find evidence of an association between the log10 concentration of ruminant markers in ruminant-marker-positive samples and sampling visit (*p* < 0.001). On average, the log10 concentration of ruminant markers was 0.72 log10 (95% CI = −1.05, −0.38; *p* < 0.001) and 1.01 log10 (95% CI = −1.43, −0.59; *p* < 0.001) lower on sampling visits two and three compared to sampling visit one, which is similar to the pattern observed for FIBs. The relationship between MST marker detection and physical-chemical water quality parameters are reported in Table S1.

### 3.4. Associations Between General Fecal Indicators and MST Markers

Univariate GLMMs were used to assess (i) the association between FIBs and MST markers, and (ii) if the relationship between FIBs and MST markers was different at urban and rural sites. Log10 fecal coliform levels were not associated with the concentration of either human (*p* = 0.428) or ruminant (*p* = 0.197) markers, or with the detection of human (*p* = 0.503) or ruminant markers (*p* = 0.127; Table 4). Moreover, models that included an interaction between land use, and either log10 marker concentration (*p* = 0.320), human marker detection (*p* = 0.129), ruminant marker concentration (*p* = 0.188), and ruminant marker detection (*p* = 0.155) did not fit the data better according to likelihood ratio tests. This suggests that the relationship between fecal coliform levels and MST markers did not differ between urban and rural sites, and that overall there was no association between MST markers and fecal coliform concentration (Table 4).

Log10 *Enterococcus* concentration was associated with both the detection (Effect Estimate = 0.4; 95% CI = 0.0,0.7; *p* = 0.050) and log10 concentration (Effect Estimate = 0.2; 95% CI = 0.02, 0.4; *p* = 0.027) of human markers. However, this association was not significantly different between rural and urban sites assessed by likelihood ratio test of inclusion of an interaction term (*p* = 0.278 for the model including log10 human marker concentration; *p* = 0.743 for the model including human marker detection). Log10 *Enterococcus* concentration was not associated with the detection (*p* = 0.620) or log10 concentration of ruminant markers (*p* = 0.514; Table 3). Models that included an interaction between land use, ruminant marker concentration, and ruminant marker detection did not fit the data better according to likelihood ratio tests (Table 3). This suggests that the relationship between *Enterococcus* concentration and MST markers did not differ between urban and rural sites (Table 3). The log10 concentration of fecal coliforms was not associated with the log10 concentration of Enterol1 (*p* = 0.91) according to a GLMM. Similarly, the log10 concentration of human markers were not associated with the log10 concentration of ruminant markers (*p* = 0.914) or the detection of ruminant markers (*p* = 0.546) according to GLMM.

## 4. Discussion

In this study, we incorporated validated MST methods into routine dry-weather monitoring to identify the major sources of contamination, which has been difficult using only traditional cultivation-based methods. While ruminant markers were occasionally identified in urban areas, we found that high levels of ruminant contaminants upstream were not present at downstream, urban locations, suggesting that these contaminants may have been significantly degraded, diluted, or deposited during in-stream transport. Importantly, this suggests that ruminant contaminants are likely not major contributors to the high levels of observed FIB in urban areas during dry weather. Low levels of ruminant markers in urban areas could be explained by transport from upstream and/or contributions from urban deer populations, which average 28 individuals/mi^2^ in some parts of the watershed [31].

Somewhat expectedly, our findings indicate that human markers strongly impacted urban reaches, and that *Enterococcus* marker concentrations were associated with human marker levels and with urban areas. Through 2018, over $660 million has been invested to reduce the input of fecal contamination to Onondaga Lake, $451 million of which has been targeted toward CSO abatement in tributaries, mainly by increasing stormwater storage capacity [19]. However, our observation that high levels of human contaminants consistently dominate urban reaches even in dry weather suggests that perhaps more resources could be directed toward persistent sources of human contaminants, such as faulty infrastructure or illicit discharges. Previous research [32] and subsequent efforts to pinpoint contaminants by measuring fecal indicator bacteria arrived at a similar conclusion [28]; however, the results from the main stem of Onondaga Creek were not always easy to interpret due to the possibility that high levels of FIB might have been transported from upstream [3,28,29]. Taken together, the MST results and prior FIB analysis provide strong evidence that weather-independent, human-derived contamination has a substantial impact on Onondaga Creek’s history of noncompliance with water quality standards.

Ruminant contaminant sources, most likely originating from agricultural runoff, dominate at and upstream of site OC4, although human markers were also detected at the two upper-most sites, OC1 and OC2. The observation that ruminant markers increased in tandem with fecal coliforms from site OC1 to OC2 on both July 6 and 20, while human markers only increased slightly, further supports the idea that ruminant sources are the major source of fecal coliforms entering the stream between these two sites. Moreover, on July 20, ruminant marker levels at OC2 were 10-fold higher than in any other sample collected as part of this study. Therefore, any future mitigation efforts to reduce fecal contamination upstream of OC2 may be most effective by targeting ruminant sources.

Unexpectedly, although we identified a significant, positive association between human markers and urban sites, we also observed high levels of human markers at the most upstream site, OC1, on two of the three sampling dates. Presumably, failing septic tanks or other on-site human waste containment structures are responsible for these upstream detections of human markers. Site OC1 is immediately next to several homes on septic. It is important to note, however, that the highest human marker concentration in a rural sample was five times lower than the highest human marker concentration in an urban sample.

Differential decay between cultivation-based fecal indicators and molecular markers complicates the interpretation of MST data [33]. The relatively high variability of fecal coliforms in our study and, in particular, the abrupt decreases in concentrations from site-to-site compared to *Enterococcus* molecular markers (e.g., OC3 to OC4 on Jul 6) are likely due to the relatively rapid decay of cultivable indicators upon exposure to sunlight [33]. If true, this observation suggests that cultivable indicators may be better indicators of where fecal contaminants enter stream systems. In contrast, slow-decaying *Enterococcus* molecular markers may be governed more by mass transfer and thus mirror the transport of fecal contaminants within a stream system. Based on this understanding of fecal coliform and MST marker dynamics post-contamination, the pattern of fecal contamination observed here indicates repeated introduction of fecal contamination into the Onondaga Creek throughout its length (as evidenced by large spikes in fecal coliform levels). Similarly, the sustained high concentrations of *Enterococcus* markers, with the highest levels occurring in downstream sites, suggests the accumulation of fecal contaminants as water moves through the watershed and/or continuous introduction of high levels of *Enterococcus* markers into the watershed throughout its reach. The fact that spikes in fecal coliform concentration (e.g., on July 6 and 20 at OC2; July 6 at OC17, OC11, and OC7) correspond to concomitant increases in MST markers qualitatively support this hypothesis. However, future research is needed to fully investigate differences in the dynamics of cultivable versus molecular indicators at this site, and how this should affect interpretation of paired FIB and MST data.

In summary, MST methods pointed to specific contaminant sources that pollute specific reaches of the periurban stream, Onondaga Creek, and allowed more specific recommendations for improving water quality and safety in the area. Although a larger study that extends into higher-order tributaries and across multiple seasons, especially spring, would help translate our findings to other sites, it is likely that similar dynamics to those observed here also occur in streams that transition from largely agricultural to urban land uses. The MST study described herein, which was done relatively cheaply, appears to be a cost-effective way to inform management strategy and calls into question the overwhelming focus on CSO events as the central means of reducing fecal bacteria exceedances.

## Supplementary Material

Maximum Value Per Site

MicrobialWaterQuality

## Figures and Tables

**Table 1.** Microbial source-tracking assays used in this study. Concentrations for each primer and probes are listed.

Assay Name	Source Target	Primer Conc. (nM)	Probe Conc. (nM)	Reference
HF183	Human	1000	80	[10]
Rum2Bac	Ruminant	300	100	[12]
DG3	Canine	1400	100	[15]
Entero1	*Enterococcus*	1000	100	[21,22]

**Table 2.** Summary of microbial and physical-chemical water quality for each site during the study period.

					Geometric Mean (Min.-Max.) ^2^				Mean (Min. Max.)				
Site	Lat.	Land Use Class.	Class ^1^	Fecal Coliforms (CFUs/100 mL)	Enterol1 (markers/100 mL)	HF183 (markers/100 mL)	Rum2Bac (markers/100 mL)	Conductivity (μS/cm)	Dissolved Oxygen (mg/L)	pH	Salinity (ppt)	Turbidity (NTUs)	Water Temperature (°C)
OC1	42.82	Rural	C	140 (27–460)	3061 (2235–5437)	NA (990–1039)	NA (320–1366)	996 (507–1484)	10.2 (9.6–10.8)	8.23 (8.18–8.28)	0.5 (0.25–0.75)	29.9 (8.2–51.6)	18.3 (17.4–19.2)
OC2	42.90	Rural	C	1633(454–4800)	6611 (3286–24694)	NA (1118–1399)	2807 (855–20326)	1202 (951–1618)	8.9 (8–9.5)	8.13 (8.1–8.16)	0.6 (0.47–0.82)	50.2 (40.3–66.3)	19.1 (15.9–21.6)
WB1	42.93	Rural	C(T)	NA (127–145)	5679 (3895–9363)	–	–	2671 (645–6663)	8.6 (8.2–8.9)	8.06 (8.0–8.1)	0.32 (0.31–0.34)	25.3 (12.7–34.5)	21.3 (19.3–22.6)
OC3	42.94	Rural	C	2638 (2000–3400)	NA (3687–13545)	–	NA (684–2123)	933 (828–1144)	8.7 (8.1–9.2)	8.1 (8.06–8.12)	0.46 (0.41–0.57)	59 (25.6–110.4)	20.2 (18.1–21.4)
OC4	42.99	Urban	B	189 (18–2600)	3682 (2192–5327)	–	NA (314–640)	1010 (848–1132)	9.3 (8.5–9.9)	8.12 (8.11–8.13)	0.5 (0.42–0.56)	29.7 (10.7–51.2)	20.1 (18.5–21)
OC14	43.00	Urban	B	372(118–2400)	6689 (2807–20209)	NA (825–825)	–	1030 (860–1174)	8.9 (8.1–9.5)	8.14 (8.13–8.16)	0.51 (0.42–0.59)	29.8 (8.4–55.2)	19.9 (18.2–20.9)
OC15	43.07	Urban	B	380 (127–1600)	10283 (3014–50474)	3942 (1839–7364)	–	1134(954–1301)	9.1 (7.9–9.7)	7.94 (7.88–7.99)	0.56 (0.47–0.65)	34.9 (5.7–62.9)	18.7 (17.4–19.9)
OC17	43.03	Urban	B	818 (91–3300)	9970 (4249–29040)	2748 (2141–3313)	NA (294–294)	1194 (1026–1389)	8.9 (7.8–9.7)	7.93 (7.87–7.96)	0.6 (0.51–0.7)	36.2 (5.5–82.6)	17.9 (16.5–19.1)
OC7	43.05	Urban	C	779 (91–3000)	12571 (6199–36643)	2259 (1949–2724)	NA (696–696)	1225 (1050–1459)	8.6 (7.8–9.4)	7.9 (7.86–7.92)	0.61 (0.52–0.74)	42.9 (5.3–93.6)	17.9 (16.4–19.4)
OC11	43.06	Urban	C	802 (364–2600)	5832 (4335–6998)	2728 (1955–3316)	–	3013 (2200–4189)	8.5 (7.8–9.3)	7.84 (7.75–7.9)	1.58 (1.13–2.24)	18.3 (5.1–27.8)	17.9 (16.3–19.6)

1 Primary uses for Class B waters are primary and secondary contact recreation and fishing. Primary uses for Class C waters are fishing. “(T)” indicates trout standards also apply. Classes B, C, and C(T) all have the same fecal coliform standard of a geometric mean of 200 CFU/100 mL calculated from a minimum of five samples per month.

2 Geometric means were not calculated for sites with less than three values within the limit of detection (“NA”).

“-” indicates all three samples were below the limit of detection.

**Table 3.** Associations of microbial water quality parameters, FIB, and MST marker concentrations with latitude, land use, and sampling visit in Onondaga Creek.

	Effect Estimate (95% Confidence Interval; *p*-Value)^1^			
Outcome	Latitude ^2^	Land Use ^3^	Sampling Visit^4^	
			7/20/15	8/3/15
Log10 Conductivity (μS/cm)	1.16 (−0.14, 2.47; 0.082)	0.08 (−0.12, 0.28; 0.407)	−0.12 (−0.31, 0.07; 0.211)	0.03 (−0.16, 0.22; 0.759)
Dissolved Oxygen (mg/L)	−0.25 (−0.42, −0.07; 0.008)	−0.29 (−1.01, 0.42; 0.421)	1.29 (1.00, 1.58; <0.001)	1.10 (0.82, 1.38; <0.001)
pH	−1.46 (−2.11, −0.81; <0.001)	−0.15 (−0.29, −0.02; 0.030)	−0.02 (−0.6, 0.02; 0.362)	−0.04 (−0.08, 0.00; 0.033)
Water Temperature (°C)	−2.92 (0–14.82, 8.98; 0.631)	−0.79 (−2.42, −2.36; 0.341)	−3.04 (−3.73, −2.37; <0.001)	−0.76 (−142, −0.11; 0.024)
Log10 Salinity (ppt)	1.51 (0.20, 2.81; 0.024)	0.18 (−0.02, 0.38; 0.083)	−0.03 (−0.10, 0.04; 0.394)	0.14 (0.07, 0.21; <0.001)
log10(Turbidity) (NTU)	−1.24 (−2.90, 0.42; 0.144)	−0.21 (−0.43, 0.01; 0.065)	−0.22 (−0.50, 0.05; 0.112)	−0.62 (−0.88, −0.35; <0.001)
Log10 Entero1/100 mL	2.19 (−0.32, 4.70; 0.087)	0.36 (0.02, 0.70; 0.041)	−0.55 (−0.96 −0.14; 0.009)	−0.73 (−1.14, −0.32; <0.001)
Log10 Fecal Coliforms/100 mL	2.06 (−2.67, 6.78;0.394)	0.08 (−0.62, 0.79; 0.823)	−0.56 (−1.12 −0.01; 0.048)	−0.69 (−1.24, −0.13; 0.016)
Human Marker Detection	– ^5^	55.98 (32.54, 79.41; <0.001)	−1.91 (−7.04, 3.23; 0.467)	−5.91 (−20.04, 8.22; *p* = 0.413)
Log10 HF183/100 mL	2.17 (0.38, 3.96; 0.018)	0.37 (0.04, 0.69; 0.030)	−0.01 (−0.18, 0.17; 0.947)	−0.12 (−0.32, 0.09; 0.271)
Ruminant Marker Detection	– ^5^	−1.92 (−4.27, 0.43; 0.110)	0.00 (−2.12, 2.12; 1.000)	−0.61 (−2.81, 1.59; 0.587)
Log10 Rum2Bac/100 mL	−1.35 (−6.00, 3.29; 0.569)	−0.28 (−0.95, 0.39; 0.416)	−0.72 (−1.05, −0.38; <0.001)	−1.01 (−1.43, −0.59; <0.001)

1 For models where the outcome is binary, the coefficient is the log odds (e.g., the change in the log odds of detecting the human marker in urban land use compared to rural). For models where the outcome is continuous, the coefficient is the effect estimate (e.g., the change in pH in urban land use compared to rural).

2 Results are from univariate models where site visit and latitude were included as fixed effects, and site was included as a random effect. Results for latitude are reported here since it was the primary factor of interest, and visit was only included to account for pseudoreplication.

3 Results are from univariate models where site visit and landuse (as a categorical variable with two levels, urban and rural, with rural being the reference-level) were included as fixed effects, and site was included as a random effect. Results for land use are reported here since it was the primary factor of interest, and visit was only included to account for pseudoreplication.

4 Results are from univariate models where site visit was included as a fixed effect and site was included as a random effect. Note sampling visit 1 (on 7/6/15) was the reference level.

5 Due to quasi-separation of latitude by human and ruminant marker detection, a model could not be developed for these two outcomes (see Figure S1).

**Table 4.** Association between microbial water quality parameters, including fecal indicator bacterial concentrations and MST marker concentrations, in Onondaga Creek.

Outcome	Effect Estimate (95% Confidence Interval; *p*-Value) ^2^				
	Log10 Enterol^1^ Concentration	Log10 Human Marker Concentration	Human Marker Detection	Log10 Ruminant Marker Concentration	Ruminant Marker Detection
Log10 Fecal Coliform Concentration	0.03 (−0.52, 0.59; 0.913)	0.13 (−0.19, 0.45; 0.428)	0.21 (−0.40, 0.82;0.503)	−0.23 (−0.58, 0.12; 0.197)	−0.42 (−0.97, 0.12; 0.127)
Log10 Entero1 Levels Concentration	–	0.20 (0.02, 0.4; 0.03)	0.35 (0.00, 0.70; 0.05)	0.08 (−0.19, 0.32; 0.51)	0.10 (−0.28, 0.48; 0.62)
Log10 Ruminant Marker Concentration	–	−0.02 (−0.323 0.29; 0.91)	−0.02 (−0.56, 0.53; 0.95)	−	−
Log10 Human Marker Concentration	–	–	–	–	−0.11 (−0.36, 0.14;0.38)
Human Marker Detection	–	–	–	–	

1 Results are from separate univariate models where site visit and the given microbial water quality parameter were included as fixed effects, and site was included as a random effect. Results for the given microbial water quality parameter are reported here since it was the primary factor of interest, and visit was only included to account for pseudoreplication.

2 For models where the outcome is binary, the coefficient is the log odds (e.g., the change in the log odds of detecting the human marker for a one unit increase in fecal coliforms). For models where the outcome is continuous, the coefficient is the effect estimate (e.g., the change in human marker concentration for a unit increase in fecal coliforms).
