# Supplementary material for: Microbial Source-Tracking Reveals Origins of Fecal Contamination in a Recovering Watershed
Source: Water (Basel). Author manuscript; Available in PMC 2020 Jun 25. (PMC7316189; doi:10.3390/w11102162)
Supplement: Maximum Value Per Site [file NIHMS1583650-supplement-Maximum_Value_Per_Site.html]

leaflet
